# The effects and safety of PD‐1/PD‐L1 inhibitors on head and neck cancer: A systematic review and meta‐analysis

**DOI:** 10.1002/cam4.2510

**Published:** 2019-08-22

**Authors:** Bi‐Cheng Wang, Ru‐Bo Cao, Pin‐Dong Li, Chen Fu

**Affiliations:** ^1^ Cancer Center Union Hospital Tongji Medical College Huazhong University of Science and Technology Wuhan China; ^2^ Department of Dermatology The First Hospital of Wuhan Wuhan China

**Keywords:** checkpoint inhibitor, head and neck cancer, human papillomavirus, PD‐1, PD‐L1

## Abstract

**Background:**

Inhibitors of programmed cell death‐1 (PD‐1) and its ligand (PD‐L1) have been increasingly used in head and neck cancer therapy and reported to improve the outcomes with an acceptable safety profile. This systematic review and meta‐analysis was conducted to assess the benefit and risk of PD‐1/PD‐L1 inhibitors in patients with head and neck cancer.

**Method:**

The PubMed, Cochrane Library, EMBASE and Web of Science databases were systematically searched to find potentially eligible studies up to May 30, 2019. Primary outcomes were overall survival (OS), progression‐free survival (PFS), objective response rate (ORR), disease control rate (DCR) and adverse events.

**Results:**

Overall, this analysis consisted of nine eligible studies, with two randomized controlled trials and seven single arm trials. In the treatment of recurrent or metastatic head and neck cancer, PD‐1 inhibitors showed significantly lower relative risk of death than standard‐of‐care therapy (odds ratio [OR] = 0.60, 95% confidence interval [CI]: 0.44‐0.82, *I* ^2^ = 0%, *P* = .001). Programmed cell death‐1 inhibitors also decreased the risk of disease progression, however, there was no statistically significant difference of PFS between the treatments (OR = 0.69, 95% CI: 0.48‐1.01, *I* ^2^ = 0%, *P* = .05). Subgroup analysis showed that human papillomavirus (HPV) positive patients had higher response rates than HPV negative patients in PD‐1/PD‐L1 inhibitors‐treated population (ORR: 18.8% vs 12.2%; DCR: 42.8% vs 34.4%). The most common any‐grade and grade ≥3 treatment‐related adverse events were fatigue (14.7%, 95% CI: 12.3%‐17.1%) and aspartate aminotransferase increased (1.6%, 95% CI: 0.3%‐2.9%), respectively.

**Conclusion:**

Programmed cell death‐1 inhibitors prolonged OS in comparison with standard‐of‐care therapy in recurrent or metastatic head and neck cancer patients. Human papillomavirus positive patients were superior to HPV negative patients in the treatment of PD‐1/PD‐L1 inhibitors. More phase III randomized controlled trials are warranted to confirm our findings.

## Graphical abstract

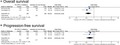

## 1 INTRODUCTION

Patients with recurrent or metastatic squamous cell carcinoma of the head and neck have a poor prognosis (a median survival of 6 months or less) and few treatment options.^1^, ^2^, ^3^ Based on the EXTREME trial, the current first‐line standard for recurrent or metastatic head and neck cancer is a triple association of a platinum‐based doublet chemotherapy and cetuximab.^2^ However, the efficacy of such palliative chemotherapy was limited.^4^ In recently KEYNOTE‐048 clinical trial, combining an anti‐programmed cell death‐1 (PD‐1) agent + platinum + 5‐fluorouricil was recommended as a frontline treatment for patients with recurrent or metastatic head and neck squamous cell carcinoma compared to the EXTREME regimen.^5^, ^6^, ^7^

High mutational burden owing to tobacco use, alcohol consumption, or human papillomavirus (HPV) expression might contribute to immunogenicity in head and neck cancer.^8^, ^9^, ^10^, ^11^ Nevertheless, overexpression of PD‐1 ligand (PD‐L1) could protect cancer cells from tumor‐specific T cells.^12^ Because tumor‐related regulation of the PD‐1/PD‐L1 axis might lead to evade immune surveillance, and cancer cells expressing PD‐L1 could reduce T‐cell effector activity and terminate immune reactions.^13^, ^14^

Fortunately, anti‐PD‐1 and anti‐PD‐L1 agents have revolutionized head and neck cancer therapy.^15^, ^16^, ^17^ To date, three PD‐1 inhibitors (pembrolizumab, nivolumab and cemiplimab) and three PD‐L1 inhibitors (atezolizumab, durvalumab and avelumab) have been approved by the US Food and Drug Administration (FDA). Blocking the PD‐1/PD‐L1 signaling pathway with monoclonal antibodies might be an effective means of restoring immune surveillance and T cell‐mediated antitumor immunity.^16^

Up to now, many clinical trials have reported the benefits and safety of PD‐1/PD‐L1 antagonists for head and neck cancer. Most of the trials found that blockage of PD‐1/PD‐L1 might improve clinical outcomes and be well tolerated. However, there are still some controversies. For instance, it is unknown whether the PD‐1/PD‐L1 inhibitors actually prolong the overall survival (OS) or progression‐free survival (PFS) and whether the HPV status is a predictive factor of efficacy for PD‐1/PD‐L1 targeted therapy in head and neck cancer.

Overall, we conducted this systematic literature review and meta‐analysis to integrate the results of current knowledge and to evaluate the toxicity of PD‐1/PD‐L1 inhibitors in head and neck cancer.

## 2 METHODS

### 2.1 Search strategy and study selection

This meta‐analysis was conducted followed the Preferred Reporting Items for Systematic Reviews and Meta‐analyses guideline (PRISMA).^18^

The search was done in the electronic databases PubMed, Cochrane Library, Web of Science, and EMBASE to identify all relevant records until 30 May 2019. Additionally, search terms included “nivolumab or pembrolizumab or cemiplimab or atezolizumab or durvalumab or avelumab or PD‐1 inhibitor or PD‐L1 inhibitor”, “head and neck cancer or head and neck or head and neck neoplasm or head and neck tumor or head and neck carcinoma”, and “trial or clinical trial or randomized clinical trial or randomized controlled trial”. We also manually searched the references of relevant published studies and reviews articles for more eligible trials. The search results were uploaded into a citation database program for further review (EndNote: http://www.endnote.com/).

Studies eligible for inclusion met all of the following criteria: (a) prospective clinical trials in patients with head and neck cancer; (b) for single arm trials, participants were treated with only an anti‐PD‐1/PD‐L1 agent; (c) for controlled clinical trials, participants in the control arm were treated with standard‐of‐care therapy; (d) antitumor activity and safety data was included; (e) trials were published in English. We excluded conference abstracts due to the absence of safety data and the increase in heterogeneity. For multiple publications that were identified reporting on the same clinical study, the one with the most complete publication data was eligible. Any discrepancies were resolved by discussion.

### 2.2 Data extraction

Detailed reviews of full‐text articles regarding study design, baseline characteristics, outcomes, and toxicities were performed by BW and CF independently. The trial name, publication year, study design, number of patients, number of male patients, PD‐L1 expression, HPV status, anti‐PD‐1/PD‐L1 agent used, dosing schedule, OS, PFS, objective response rate (ORR), disease control rate (DCR), median duration of response, and safety data (number of any‐grade and grade ≥3 treatment related adverse events) reporting in the articles and Supplementary materials were obtained from each included study.

### 2.3 Statistical analysis

Data from randomized controlled trials (OS and PFS) and PD‐1/PD‐L1 arm (ORR and DCR) was assessed by odds ratio (OR) and 95% confidence interval (CI). The subgroup analyses of OS were assessed by hazard ratio (HR) and 95% CI because of the absence of individual data. The above‐mentioned meta‐analyses were conducted using RevMan version 5.3 software (Cochrane Collaboration's Information Management System). If the heterogeneity test showed no statistical significance (*P* ≥ .10, *I* ^2^ ≤ 50%), a fixed‐effects model was used. Otherwise, a random‐effects model was applied; *P* < .05 was considered as statistically significant outcomes.

Pooled effect sizes of ORR, DCR, and treatment‐related adverse events were done using STATA statistical software, version 14.0. Random‐effects models were applied in these analyses due to the single arm data. Statistical heterogeneity among the studies was tested by the Cochran Q chi‐square test and *I* ^2^ statistic percentages. *I* ^2^ < 50% or *P* > .10 was defined as low heterogeneity, otherwise was high heterogeneity. Egger's test was displayed to evaluate latent publication bias for small‐study effects.

## 3 RESULTS

### 3.1 Eligible studies and characteristics

**Figure 1:**
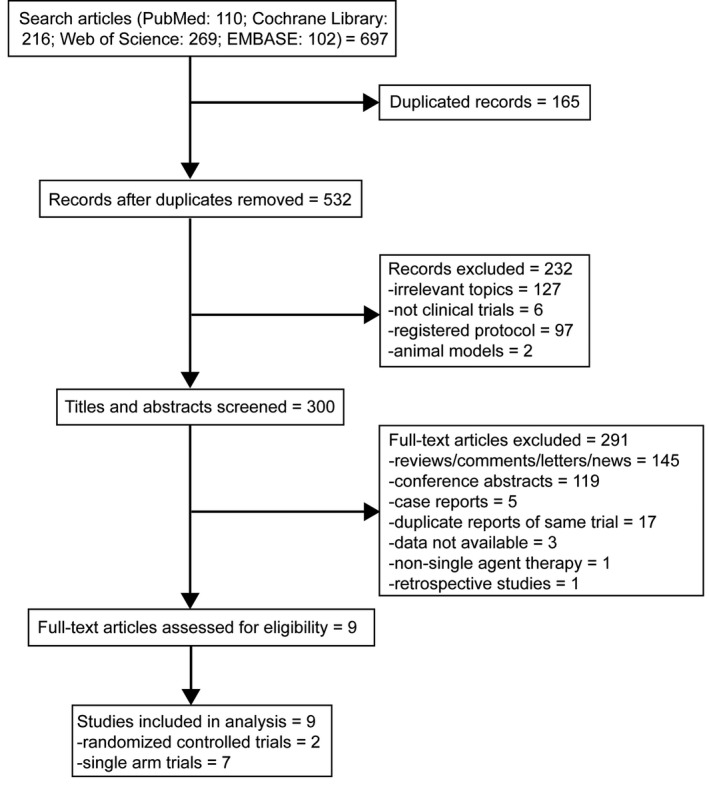
Flow chart of systematic literature search

Literature search and review of reference lists identified 697 relevant articles. After screening and eligibility assessment, we included in the systematic review and meta‐analysis a total of nine clinical trials involving the group (n = 1018) of patients treated with PD‐1/PD‐L1 inhibitors and the group (n = 369) receiving standard‐of‐care treatment, comprising two randomized controlled trial and seven single arm trials (Figure 1).^19^, ^20^, ^21^, ^22^, ^23^, ^24^, ^25^, ^26^, ^27^ The PD‐1/PD‐L1 inhibitors used included pembrolizumab (n = 4), nivolumab (n = 1), cemiplimab (n = 0), atezolizumab (n = 1), avelumab (n = 0), and durvalumab (n = 3). All participants in nine studies were diagnosed with recurrent or metastatic head and neck cancer. Head and neck cancers were in the oropharynx, tongue, oral cavity, nasal cavity, hypopharynx, larynx, pharynx, nasopharynx, and other or unknown regions in the head and neck. The characteristics of the nine eligible studies were described in Table 1, and the summary of outcomes was presented in Table 2.

**Table 1:** Characteristics of the eligible studies in the meta‐analysis

Study	Year	Design	No. patients	Male, no. (%)	PD‐L1+, no. (%)	HPV+, no. (%)	HPV‐, no. (%)	Age (mean, range)	Drug	Dose
KEYNOTE‐012	2016	Phase Ib	60	49 (81.7)	60 (100)	23 (38.3)	37 (61.7)	63 (20‐83)	Pembrolizumab	10 mg/kg, iv, q2weeks
KEYNOTE‐028	2017	Phase Ib	27	21 (77.8)	27 (100)	NR	NR	52 (18‐68)	Pembrolizumab	10 mg/kg, iv, q2weeks
KEYNOTE‐040	2018	Phase III	Pem: 247 Sta: 248	207 (83.8) 205 (82.7)	NR	NR	NR	60 (55‐66) 60 (54‐66)	Pembrolizumab	200 mg, iv, q3weeks
KEYNOTE‐055	2017	Phase II	171	138 (80.7)	140 (81.9)	37 (21.6)	131 (76.6)	61 (33‐90)	Pembrolizumab	200 mg, iv, q3weeks
CheckMate 141	2016	Phase III	Niv: 240 Sta: 121	197 (82.1) 103 (85.1)	88 (36.7) 61 (50.4)	63 (26.2) 29 (24.0)	50 (20.8) 36 (29.8)	59 (29‐83) 61 (28‐78)	Nivolumab	3 mg/kg, iv, q2weeks
PCD4989g	2018	Phase I	32	27 (84.4)	NR	13 (40.6)	12 (37.5)	62 (32‐78)	Atzolizumab	15 mg/kg, 20 mg/kg, or a 1200‐mg fixed dose, iv, q3weeks
CONDOR	2018	Phase II	67	54 (80.6)	NR	18 (26.9)	NR	62 (23‐82)	Durvalumab	10 mg/kg, iv, q2weeks
HAWK	2019	Phase II	112	80 (71.4)	112 (100)	34 (34.3)	65 (65.7)	60 (24‐84)	Durvalumab	10 mg/kg, iv, q2weeks
MedImmune	2019	Phase I/II	62	53 (85.5)	NR	25 (40.3)	25 (40.3)	57 (24‐96)	Durvalumab	10 mg/kg, iv, q2weeks

Abbreviations: Niv, nivolumab group; NR, not reported; PD‐L1+, PD‐L1 > 1% in tumor and immune; Pem, pembrolizumab group; Sta, standard‐of‐care group.

**Table 2:** Summary of outcomes in the selected studies

Study	Median follow‐up	Median duration of response	Median PFS	Median OS
KEYNOTE‐012	14.0 mo (interquartile range (IQR), 4.0‐14.0)	12.4 mo (95% CI: 3.0‐not reached)	2.0 mo (95% CI: 2.0‐4.0)	13.0 mo (95% CI: 5.0‐not reached)
KEYNOTE‐028	20 mo (range, 2.2‐26.8)	17.1 mo (range, 4.8‐22.1+)	6.5 mo (95% CI: 3.6‐13.4)	16.5 mo (95% CI: 10.1‐not reached)
KEYNOTE‐040	7.5 mo (IQR, 3.4‐13.3)	Pem: 18.4 mo (95% CI: 5.8‐18.4) Sta: 5.0 mo (95% CI: 3.6‐18.8)	3.5 mo (95% CI: 3.1‐4.4) 4.8 mo (95% CI: 4.1‐5.7)	8.4 mo (95% CI: 6.4‐9.4) 6.9 mo (95% CI: 5.9‐8.0)
KEYNOTE‐055	9.0 mo (range, 7.0‐17.0)	8 mo (range, 2.0‐12.0)	2.1 mo (95% CI: 2.1‐2.1)	8.0 mo (95% CI: 6.0‐11.0)
CheckMate 141	5.1 mo (range, 0‐16.8)	Pem: NR Sta: NR	2.0 mo (95% CI: 1.9‐2.1) 2.3 mo (95% CI: 1.9‐3.1)	7.5 mo (95% CI: 5.5‐9.1) 5.1 mo (95% CI: 4.0‐6.0)
PCD4989g	NR	7.4 mo (range, 2.8‐45.8)	2.6 mo (range, 0.5‐48.4)	6.0 mo (range, 0.5‐51.6+)
CONDOR	6.0 mo (range, 0.3‐18.0)	NR	1.9 mo (95% CI: 1.8‐2.8)	6.0 mo (95% CI: 4.0‐11.3)
HAWK	6.1 mo (range, 0.2‐24.3)	10.3 mo	2.1 mo (95% CI: 1.9‐3.7)	7.1 mo (95% CI: 4.9‐9.9)
MedImmune	40.3 mo (range, 1.4‐49.2)	12.4 mo (range, 3.5‐20.5+)	1.4 mo (95% CI: 1.4‐1.5)	8.4 mo (95% CI: 5.7‐12.3)

Abbreviations: 95% CI, 95% confidence interval; Niv, nivolumab group; NR, not reported; OS, overall survival; Pem, pembrolizumab group; PFS, progression‐free survival; Sta, standard‐of‐care group.

### 3.2 Overall survival

**Figure 2:**
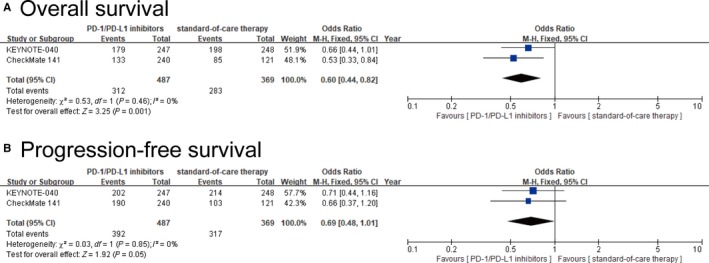
Forest plots of overall survival and progression‐free survival for the meta‐analysis. A, Forest plot of odds ratio for overall survival in head and neck patients between PD‐1/PD‐L1 inhibitors and standard‐of‐care therapy. B, Forest plot of odds ratio for progression‐free survival in head and neck patients between PD‐1/PD‐L1 inhibitors and standard‐of‐care therapy. CI, confidence interval; Fix, fixed effect analysis model; *I* ^2^, index of heterogeneity; M‐H, Mantel‐Haenszel statistical method

Data regarding OS was available from two of nine studies,^21^, ^23^ including 487 patients in the PD‐1/PD‐L1 inhibitor group and 369 patients in the standard‐of‐care therapy group. Forest plots showed that the PD‐1/PD‐L1 inhibitor group had a 40% lower risk of death compared to the standard‐of‐care therapy group (OR = 0.60, 95% CI: 0.44‐0.82, *I* ^2^ = 0%, *P* = .001) (Figure 2A).

### 3.3 Progression‐free survival

Progression‐free survival data was also available from the two studies in OS analysis. Forest plots showed that the PD‐1/PD‐L1 inhibitor group had a 31% lower risk of disease progression compared to the standard‐of‐care therapy group, but the difference was not statistically significant (OR = 0.69, 95% CI: 0.48‐1.01, *I* ^2^ = 0%, *P* = .05) (Figure 2B).

### 3.4 Overall survival in PD‐L1 positive and PD‐L1 negative subgroups

**Figure 3:**
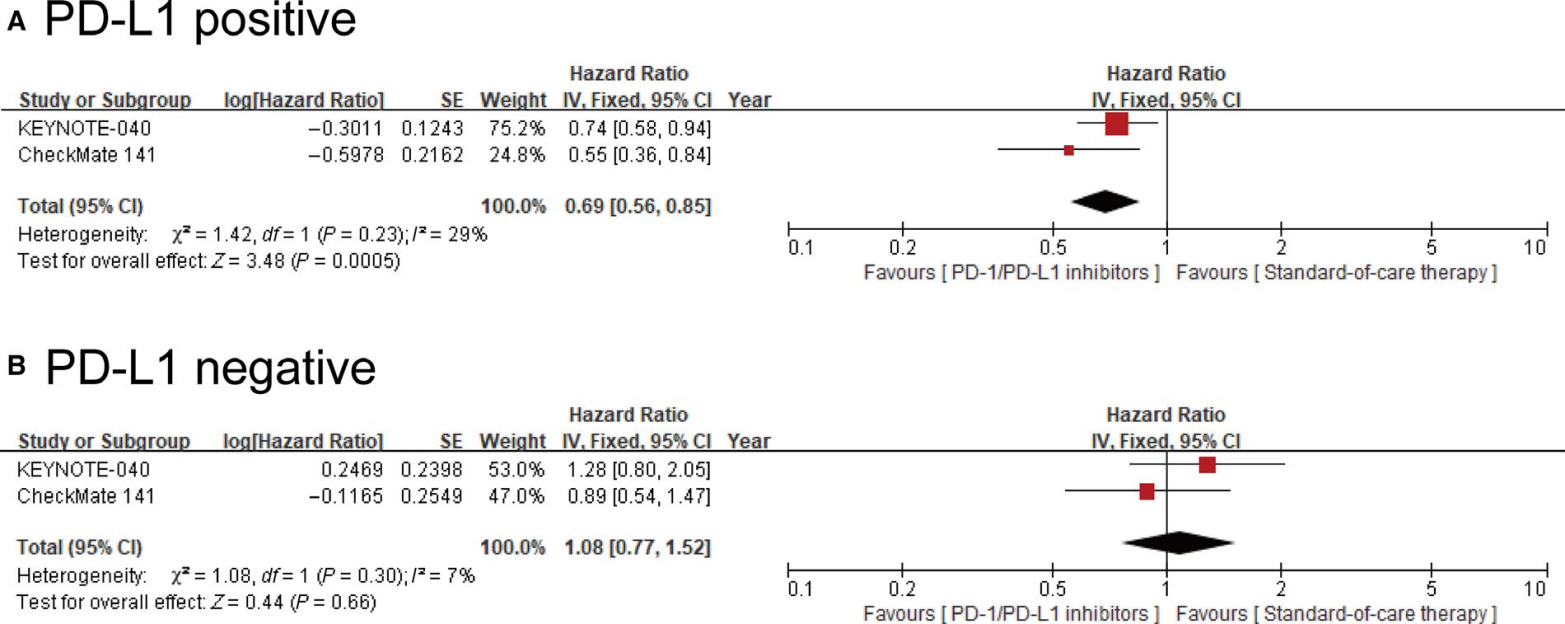
Forest plots of overall survival in PD‐L1 positive and PD‐L1 negative population. A, Forest plot of hazard ratio for overall survival in PD‐L1 positive head and neck patients between PD‐1/PD‐L1 inhibitors and standard‐of‐care therapy. B, Forest plot of hazard ratio for overall survival in PD‐L1 negative head and neck patients between PD‐1/PD‐L1 inhibitors and standard‐of‐care therapy

In the pooled analysis of OS in the subgroup of PD‐L1 positive patients, PD‐1 inhibitors‐treated patients had a risk of death that was 31% lower than the risk among patients assigned to standard‐of‐care therapy. The HR for death among patients treated with PD‐1 inhibitors vs standard‐of‐care therapy was 0.69 (95% CI: 0.56‐0.85, *I* ^2^ = 29%, *P* < .001) (Figure 3A), whereas in the subgroup of PD‐L1 negative patients, the HR was 1.08 (95% CI: 0.77‐1.52, *I* ^2^ = 7%, *P* = .66) (Figure 3B).

### 3.5 Objective response rate by single arm analysis

**Figure 4:**
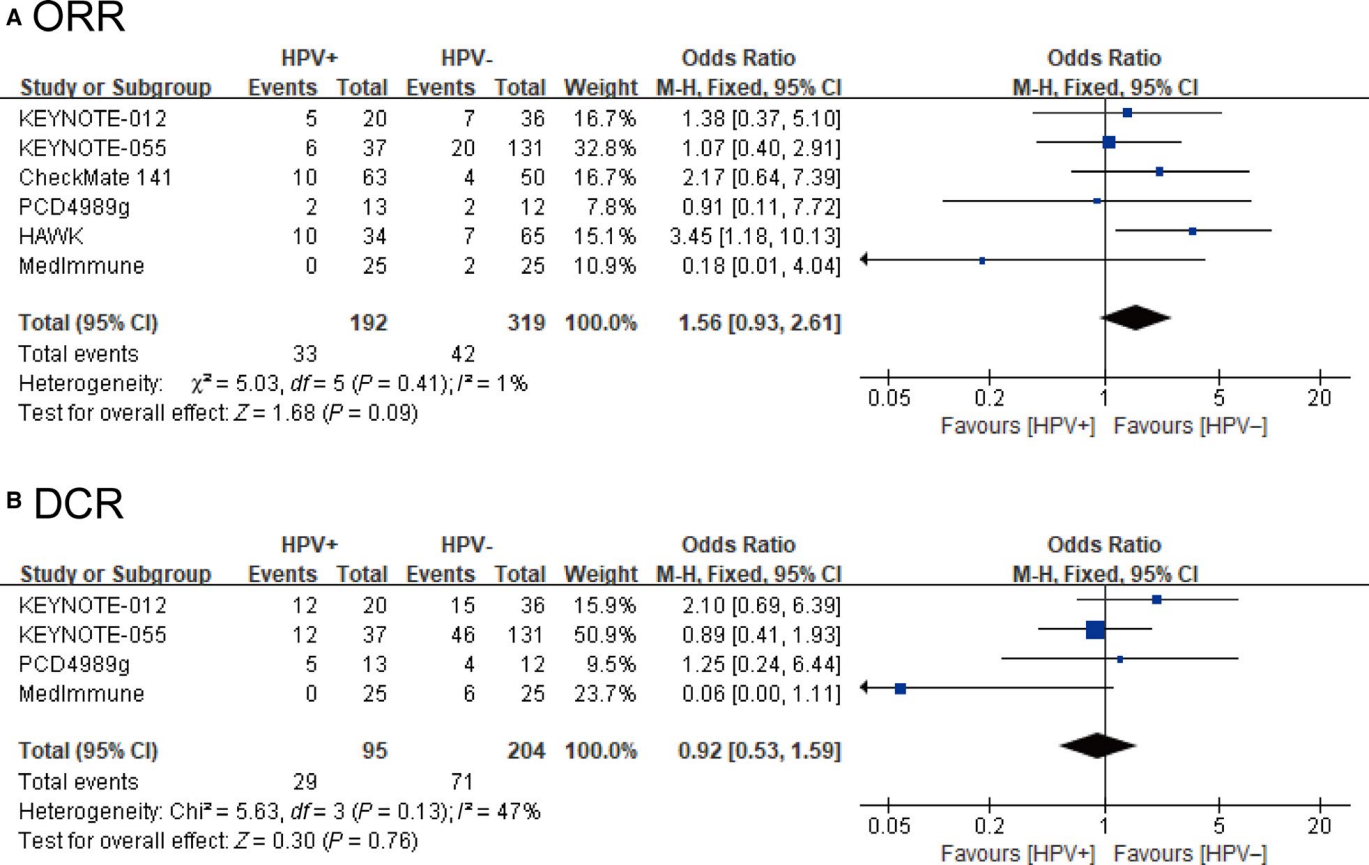
Forest plots of objective response rates and disease control rates for the meta‐analysis. A, Forest plot of odds ratio for objective response rates between human papillomavirus (HPV) positive and HPV negative head and neck cancer patients. B, Forest plot of odds ratio for disease control rates between HPV positive and HPV negative head and neck cancer patients. CI, confidence interval; *I* ^2^, index of heterogeneity; DCR, disease control rate; M‐H, Mantel‐Haenszel statistical method; ORR, objective response rate

The ORR data were available from nine studies. Pooled ORR of overall patients was 14.2% (95% CI: 11.2%‐17.2%). Subgroup analysis indicated that the pooled ORR of HPV‐positive group was 18.8% (95% CI: 12.9%‐24.6%) vs HPV‐negative group was 12.2% (95% CI: 8.6%‐15.7%) (Table 3). In six trials included both HPV‐positive and HPV‐negative groups, we analyzed the OR for ORR in PD‐1/PD‐L1 arm, involving 192 patients in the HPV‐positive group and 319 patients in the HPV‐negative group. Forest plots showed that patients in the HPV‐positive group had a 56% higher chance to achieve ORR in comparison with the HPV‐negative group. However, there was no statistically significant difference (OR = 1.56, 95% CI: 0.93‐2.61, *I* ^2^ = 1%, *P* = .09) (Figure 4A).

**Table 3:** Pooled ORR in head and neck cancer patients

Overall		HPV+		HPV−	
Study	Incidence	Study	Incidence	Study	Incidence
KEYNOTE‐012	0.214 (95% CI: 0.107‐0.322)	KEYNOTE‐012	0.250 (95% CI: 0.060‐0.440)	KEYNOTE‐012	0.194 (95% CI: 0.065‐0.324)
KEYNOTE‐028	0.259 (95% CI: 0.094‐0.425)	KEYNOTE‐055	0.162 (95% CI: 0.043‐0.281)	KEYNOTE‐055	0.153 (95% CI: 0.091‐0.214)
KEYNOTE‐040	0.146 (95% CI: 0.102‐0.190)	CheckMate 141	0.159 (95% CI: 0.068‐0.249)	CheckMate 141	0.080 (95% CI: 0.005‐0.155)
KEYNOTE‐055	0.164 (95% CI: 0.108‐0.219)	PCD4989g	0.154 (95% CI: −0.042‐0.350)	PCD4989g	0.167 (95% CI: −0.044‐0.378)
CheckMate 141	0.133 (95% CI: 0.090‐0.176)	HAWK	0.294 (95% CI: 0.141‐0.447)	HAWK	0.108 (95% CI: 0.032‐0.183)
PCD4989g	0.219 (95% CI: 0.076‐0.362)			MedImmune	0.080 (95% CI: −0.026‐0.186)
CONDOR	0.092 (95% CI: 0.022‐0.163)				
HAWK	0.162 (95% CI: 0.094‐0.231)				
MedImmune	0.065 (95% CI: 0.003‐0.126)				
Total	0.142 (95% CI: 0.112‐0.172)		0.188 (95% CI: 0.129‐0.246)		0.122 (95% CI: 0.086‐0.157)
Heterogeneity	*I* ^2^ = 42.3%, *P* = .086		*I* ^2^ = 0%, *P* = .565		*I* ^2^ = 0%, *P* = .512
Egger's test	*P* = .217		*P* = .347		*P* = .751

Abbreviations: HPV, human papillomavirus; ORR, objective response rate.

### 3.6 Disease control rate by single arm analysis

The DCR data was obtained from eight of nine studies. Pooled DCR of overall patients was 34.3% (95% CI: 25.0%‐43.6%). Subgroup analysis indicated that HPV‐positive group (incidence: 42.8%, 95% CI: 25.4%‐60.2%) had a higher DCR than HPV‐negative group (incidence: 34.4%, 95% CI: 27.9%‐40.9%) (Table 4). The four trial included both HPV‐positive and HPV‐negative groups, we analyzed the OR for DCR in PD‐1/PD‐L1 arm, involving 95 patients in the HPV‐positive group and 204 patients in the HPV‐negative group. Forest plots showed that there was no significantly difference between the two groups (OR = 0.92, 95% CI: 0.53‐1.59, *I* ^2^ = 47%, *P* = .76) (Figure 4B).

**Table 4:** Pooled DCR in head and neck cancer patients

Overall		HPV+		HPV−	
Study	Incidence	Study	Incidence	Study	Incidence
KEYNOTE‐012	0.482 (95% CI: 0.351‐0.613)	KEYNOTE‐012	0.600 (95% CI: 0.385‐0.815)	KEYNOTE‐012	0.417 (95% CI: 0.256‐0.578)
KEYNOTE‐028	0.370 (95% CI: 0.188‐0.553)	KEYNOTE‐055	0.324 (95% CI: 0.173‐0.475)	KEYNOTE‐055	0.351 (95% CI: 0.269‐0.433)
KEYNOTE‐040	0.372 (95% CI: 0.312‐0.433)	PCD4989g	0.385 (95% CI: 0.120‐0.649)	PCD4989g	0.333 (95% CI: 0.067‐0.600)
KEYNOTE‐055	0.357 (95% CI: 0.285‐0.429)			MedImmune	0.240 (95% CI: 0.073‐0.407)
PCD4989g	0.313 (95% CI: 0.152‐0.473)				
CONDOR	0.215 (95% CI: 0.115‐0.315)				
HAWK	0.523 (95% CI: 0.430‐0.615)				
MedImmune	0.129 (95% CI: 0.046‐0.212)				
Total	0.343 (95% CI: 0.250‐0.436)		0.428 (95% CI: 0.254‐0.602)		0.344 (95% CI: 0.279‐0.409)
Heterogeneity	*I* ^2^ = 86.4%, *P* < .001		*I* ^2^ = 53.3%, *P* = .117		*I* ^2^ = 0%, *P* = .513
Egger's test	*P* = .914		*P* = .647		*P* = .804

Abbreviations: DCR, disease control rate; HPV, human papillomavirus.

### 3.7 Treatment‐related adverse events

Overall, 624 (61.7%) of 1011 head and neck cancer patients from eligible studies developed at least one adverse event of any grade, and 135 (13.4%) of 1011 developed at least one grade ≥3 adverse event. The incidence of any‐grade adverse events was 61.9% (95% CI: 58.9%‐64.8%, *I* ^2^ = 0%, *P* = .258), and the incidence of grade ≥3 adverse events was 12.8% (95% CI: 10.5%‐15.0%, *I* ^2^ = 14.2%, *P* = .269) (Table 5). We integrated the data of adverse events that was extractable by at least three trials. The most frequent any‐grade adverse events were fatigue (14.7%, 95% CI: 12.3%‐17.1%), hypothyroidism (9.5%, 95% CI: 5.9%‐13.2%), rash (6.8%, 95% CI: 4.1%‐9.6%), pruritus (6.8%, 95% CI: 4.8%‐8.9%), diarrhea (6.6%, 95% CI: 5.1%‐8.1%), and nausea (6.2%, 95% CI: 3.8%‐8.5%) (Table 6). For grade ≥3 treatment‐related adverse events, aspartate aminotransferase increased (1.6%, 95% CI: 0.3%‐2.9%) was most common, followed by fatigue (1.3%, 95% CI: 0.5%‐2.1%), pneumonia (1.2%, 95% CI: 0.3%‐2.1%), and diarrhea (1.0%, 95% CI: 0.1%‐1.9%) (Table 7).

**Table 5:** Pooled analysis of any‐grade and grade ≥ 3 adverse events in head and neck cancer patients

Study	Incidence	
	Any‐grade	Grade ≥ 3
PD‐1 inhibitor		
KEYNOTE‐012	0.633 (95% CI: 0.511‐0.755)	0.167 (95% CI: 0.072‐0.261)
KEYNOTE‐028	0.741 (95% CI: 0.575‐0.906)	0.296 (95% CI: 0.124‐0.469)
KEYNOTE‐040	0.630 (95% CI: 0.570‐0.690)	0.134 (95% CI: 0.092‐0.177)
KEYNOTE‐055	0.637 (95% CI: 0.565‐0.709)	0.152 (95% CI: 0.098‐0.206)
CheckMate 141	0.589 (95% CI: 0.526‐0.652)	0.131 (95% CI: 0.088‐0.174)
Sub‐total	0.624 (95% CI: 0.590‐0.659)	0.143 (95% CI: 0.118‐0.168)
PD‐L1 inhibitor		
PCD4989g	0.656 (95% CI: 0.492‐0.821)	0.125 (95% CI: 0.010‐0.240)
CONDOR	0.631 (95% CI: 0.513‐0.748)	0.123 (95% CI: 0.043‐0.203)
HAWK	0.571 (95% CI: 0.480‐0.663)	0.080 (95% CI: 0.030‐0.131)
MedImmune	0.597 (95% CI: 0.475‐0.719)	0.097 (95% CI: 0.023‐0.170)
Sub‐total	0.602 (95% CI: 0.544‐0.661)	0.097 (95% CI: 0.061‐0.132)
Total	0.619 (95% CI: 0.589‐0.648)	0.128 (95% CI: 0.105‐0.150)
Heterogeneity	*I* ^2^ = 0%, *P* = .779	*I* ^2^ = 14.2%, *P* = .316
Egger's test	*P* = .258	*P* = .269

Abbreviations: PD‐1, programmed cell death‐1; PD‐L1, programmed cell death‐1 ligand.

**Table 6:** Analysis of any grade adverse events in head and neck cancer

Study	Incidence	95% CI
Fatigue	0.147	0.123‐0.171
Hypothyroidism	0.095	0.059‐0.132
Rash	0.068	0.041‐0.096
Pruritus	0.068	0.048‐0.089
Diarrhea	0.066	0.051‐0.081
Nausea	0.062	0.038‐0.085
AST increased	0.046	0.022‐0.070
Anemia	0.042	0.021‐0.064
Pneumonia	0.038	0.023‐0.053
Alanine aminotransferase (ALT) increased	0.037	0.015‐0.059

**Table 7:** Analysis of grade ≥ 3 adverse events in head and neck cancer

Study	Incidence	95% CI
Aspartate aminotransferase (AST) increased	0.016	0.003‐0.029
Fatigue	0.013	0.005‐0.021
Pneumonia	0.012	0.003‐0.021
Diarrhea	0.010	0.001‐0.019
Anemia	0.008	0.001‐0.014

Abbreviations: 95% CI, 95% confidence interval.

### 3.8 Publication bias

For OS and PFS, publication bias was not assessed due to the small number of studies analyzed. In the results of Egger's test based on the overall and subgroup analyses of ORR (*P* = .246 > 0.1) and DCR (*P* = .914 > 0.1), publication bias was not observed. Above all, this meta‐analysis was at low risk for reporting bias.

## 4 DISCUSSION

Most head and neck cancer patients presented with stage III–IV disease at diagnosis. Over half of the patients recurred loco‐regionally or distantly and median OS was less than a year.^2^, ^28^, ^29^ The poor outcome emphasizes the need for better treatment strategies. Moreover, head and neck cancer is an immunosuppressive tumor, with a lower lymphocyte count than those found in healthy participants.^30^ Programmed cell death‐1 ligand was found to be expressed in up to 60% of patients with head and neck cancer, leading to impaired natural killer cell activity and poor antigen‐presenting function.^30^, ^31^ Development of anti‐PD‐1/PD‐L1 agents might be a novel way to modify the standard treatment of head and neck cancer.

Thus, we conducted this meta‐analysis to estimate the antitumor activity and safety of PD‐1/PD‐L1 inhibitors in patients with head and neck cancer. The present meta‐analysis quantitatively integrated the data of published clinical trials.

In the eligible studies, only CheckMate 141 and KEYNOTE‐040 were randomized and standard‐of‐care therapy controlled clinical trials. OS and PFS data were extracted for further analyses. However, we noticed that PD‐1 inhibitors prolonged OS in head and neck cancer patients, but shortened PFS compared with standard‐of‐care therapy, regardless of pretreatments. The difference might be attributed to the inclusion of both PD‐L1 positive and PD‐L1 negative patients.^32^, ^33^, ^34^ In a phase III study, KEYNOTE‐040, the survival results also showed that pembrolizumab provided a clinically meaningful prolongation of OS, but not PFS, compared with standard‐of‐care in patients with recurrent or metastatic head and neck cancer (OS: HR 0.80, 95% CI: 0.65‐0.98, *P* = .0161; PFS: HR 0.96, 95% CI: 0.79‐1.16, *P* = .33).^35^

Immune checkpoint inhibition with monoclonal antibodies against cytotoxic T lymphocyte antigen‐4 and PD‐1/PD‐L1 by combination treatments became another new option in head and neck cancer.^36^ The ongoing EAGLE study is comparing the efficacy and safety of durvalumab as monotherapy or in combination with tremelimumab to standard‐of‐care in head and neck cancer.^37^

In CheckMate 141 trial, treatment with nivolumab resulted in longer OS than standard therapy, regardless of PD‐L1 expression or HPV status. KEYNOTE‐055 and PCD4989g studies also concluded that no association between HPV status and response to pembrolizumab or atezolizumab was detected. However, HAWK study revealed that HPV positive patients with recurrent or metastatic head and neck cancer had a numerically higher response rate and survival than HPV negative patients.

In the pooled analysis of ORR and DCR, HPV positive patients had a higher ORR (18.8% vs 12.2%) and a higher DCR (42.8% vs 34.4%) than HPV negative patients. However, in the single arm analysis, PD‐1/PD‐L1 inhibitors treated HPV positive patients showed a higher ORR, the difference was not statistically significant. Even though there was no significant difference, HPV status could also be a potential predictive biomarker in the immune checkpoint inhibitors treatments for patients with recurrent or metastatic head and neck cancer. This hypothesis will need to be tested to explain the responses observed in the subgroup based on HPV status.

All selected clinical trials reported a manageable safety profile. Nonetheless, from the standpoint of patient counseling, several results of treatment‐related adverse events should be paid more attention. Approximately, 61.9% head and neck cancer patients treated with PD‐1/PD‐L1 inhibitors in clinical trials experienced at least one adverse event of any grade, and 12.8% head and neck cancer patients had at least one grade ≥3 adverse event. Moreover, PD‐1 inhibitors had a higher incidence of any‐grade and grade ≥3 adverse events than PD‐L1 inhibitors (any‐grade: 62.4% vs 61.9%; grade ≥3:14.3% vs 12.8%). These numbers can be important to share with patients with head and neck cancer before they begin treatment with a PD‐1/PD‐L1 antagonist. Fatigue was the most common any‐grade treatment‐related adverse event (14.7%), and AST increase was the most common grade ≥3 treatment‐related adverse event (1.6%). Hypothyroidism, rash, pruritus, diarrhea, and nausea are the next most common any‐grade adverse events (>5%). Considering the potential toxicities, clinical vigilance is needed for early recognition and intervention to prevent severe complications.

### 4.1 Limitations

This meta‐analysis has several limitations. First, there were only two randomized controlled trials limiting the analysis in our study. Second, the analyses of ORR and DCR between HPV positive and HPV negative head and neck cancer patients were single arm researches. Despite these limitations, this meta‐analysis is a meaningful study of the estimates the benefits and risk of PD‐1/PD‐L1 antagonists.

In conclusion, PD‐1/PD‐L1 inhibitors appeared to be effective for treating recurrent or metastatic head and neck cancer. Moreover, our study of anti‐PD‐1 therapy indicated a superior survival in patients with PD‐L1 positive recurrent or metastatic head and neck cancer. At the same time, careful monitoring of the treatment related adverse events of anti‐PD‐1/PD‐L1 agents will be needed. More phase III randomized clinical trials are warranted to confirm our findings.

## CONFLICT OF INTEREST

None declared.

## AUTHOR CONTRIBUTIONS

Study design, data extraction, and data analysis: BW and CF; Manuscript writing and edition: BW, RC and PL.
